# Footprints To Follow

**DOI:** 10.4103/0973-1229.32146

**Published:** 2007

Some two decades ago, I was travelling in a bus for a conducted tour of some scenic spot for which organizers of annual conferences of psychiatrists take delegates. Sitting with me was the area manager of the pharmaceutical concern that, if my memory serves me right, had sponsored the tour. As the bus journey took some time, he got talking and we came to the topic of doctors and rep's visits to them.

Well, it was a chastening experience in more ways than one. Doctors always have a rather patronizing attitude towards rep's visits, which the latter know very well. But are ready to play along, nevertheless. It's a great ego massage for some of the docs to smile condescendingly as reps detail products and enjoy their sense of superiority as reps play ball sportingly. The poor guys often wait hours outside non-ac consulting rooms/clinics, uncomfortable behind tie scratchy stiff collars and still manage the easy smile when their turn comes to enter the clinic. And play ball even if some doctors call in three at a time and talk to the other two while the first is detailing his product. There are some who expect reps to come by 9 pm but they can meet the doctor only after 12 midnight, after he has finished all his patients. And one of them tells them to continue detailing their product, while he is busy counting his day's cash collection. As he finishes, the rep is supposed to quietly leave his samples, gifts etc on the table. And his departure is greeted with a gruff ‘huh’, the only concession the doctor makes to any presence beyond the important task of cash counting at hand. For the cash has to tally and the pretty receptionist has to leave, since it's already so late. And her smile as she leaves is more important than the smiling rep's detailing, samples, gifts etc. For they are to come on the table, anyway. And his smile is unconditional, hers isn't.

What struck the young consultant in me then was that it's not that reps don't understand our patronizing behaviour, but are trained (or have learnt) to ignore it as they have a job at hand. What also struck me was the fact that doctors′ idiosyncrasies, their unrealistic demands for gifts and sponsorships are tolerated but discussed in the medical rep community with open glee. Something the docs don't probably know, since the reps are always properly respectful to them. So I decided to behave myself with them always, something that reps who know me will vouch for.

Something else struck me too. As the area manager talked about the funny things that docs do, he came to one of them. His voice mellowed with respect, his face was suffused with almost a reverence. I can still remember the expression on his face as he talked about him words to this effect:

But there is one doctor who never behaves badly with us. He takes no gifts or samples or sponsorships. He talks caringly with us, even sometimes asks about our welfare and if all is fine in the family. All of us in the rep world know how good he is and we all respect him. And there is only one of this kind in the whole of Bombay.

I remembered the big stock of samples lying unused in my clinic, which I had a problem accommodating but could not also throw away. I remembered the glossy calendars that adorned the walls of my home and clinic, courtesy the same pharmaceutical of which he was the area manager. I remembered the gifts of bags, pens, clocks, wallets, ties, display items, bric-a-brac, which occupied my table and my house. I remembered the joy with which I would look as the med rep plunged into his bag after he had detailed his product and placed his samples on the table. For that last plunge meant he had something extra to offer. The magician conjuring the candy to please the child doc.

One doc had none of this.

He had said there was only one of this kind in Bombay.

Well, there was a second that moment onwards.

Ajai Singh

